# Functional and Environmental Constraints on Prey Capture Speed in a Lizard

**DOI:** 10.1093/iob/obaa022

**Published:** 2020-08-07

**Authors:** D R Adams, M E Gifford

**Affiliations:** 1 Vilonia High School, 1164 Main St, Vilonia, AR 72173, USA; 2 Department of Biology, University of Central Arkansas, 201 Donaghey Ave, Conway, AR 72035, USA

## Abstract

Movement is an important component of animal behavior and determines how an organism interacts with its environment. The speed at which an animal moves through its environment can be constrained by internal (e.g., physiological state) and external factors (e.g., habitat complexity). When foraging, animals should move at speeds that maximize prey capture while minimizing mistakes (i.e., missing prey, slipping). We used experimental arenas containing obstacles spaced in different arrays to test how variation in habitat complexity influenced attack distance, prey capture speed, and foraging success in the Prairie Lizard. Obstacles spaced uniformly across arenas resulted in 15% slower prey capture speed and 30–38% shorter attack distance compared to arenas with no obstacles or with obstacles clustered in opposite corners of the arena. Prey capture probability was not influenced by arena type or capture speed, but declined with increasing attack distance. Similarly, the probability of prey consumption declined with attack distance across arena types. However, prey consumption probability declined with increasing prey capture speed in more open arenas but not in the cluttered arena. Foraging accuracy declined with increasing speed in more open arenas, and remained relatively constant when obstacles were in closer proximity. Foraging success was primarily constrained by intrinsic properties (speed-maneuverability tradeoff) when ample space was available, but environmental conditions had a greater impact on foraging success in “cluttered” habitats. This empirical test of theoretical predictions about optimal movement speeds in animals provides a step forward in understanding how animals select speeds in nature.

## Introduction

Movement is a critical characteristic of animal behavior and regulates the ways animals interact with their environment, including avoiding predators, acquiring food, defending territories, and finding mates. Functional traits, such as locomotion, have long been of great interest to evolutionary biologists because of the suspected, and often demonstrated, relationship between performance and fitness (survival and reproductive success; Arnold 1983; Bennett and Huey 1990; Husak 2015). Traditionally, performance (i.e., ability of an organism to accomplish an ecological task using dynamic motion) has been studied by examining putative maximal capacities measured under steady-state conditions in the laboratory. However, the last couple decades have seen a significant increase in studies relating maximal performance (as measured in the laboratory) to performance actually used in nature in specific behavioral contexts—for example, evading a predator, foraging, or undisturbed locomotion (Irschick and Losos 1998; Jayne and Ellis 1998; Irschick and Jayne 1999; Irschick et al. 2005; Husak and Fox 2006; Muniz-Pagan et al. 2012). The consistent finding in these studies is that performance used in nature is typically at a level below maximal capacities and dependent on behavioral context. In lizards (the taxon in which these questions have been most frequently studied), undisturbed locomotion and foraging were typically only 30–40% of maximal sprinting capacity, whereas fleeing a predator elicited a response using a significantly greater proportion of maximal sprinting capacity, though still only 60–80% (Husak and Fox 2006). Furthermore, many animals frequently exhibit intermittent locomotion, repeated bouts of brief, sometimes strenuous, locomotor movement or behavior that is interrupted by short bouts of stasis (Gleeson and Hancock 2001). Intermittent locomotion is widespread among animals and likely characterizes most behaviors (O’Brien et al. 1990) at least partly because it reduces the overall energetic cost of the activity (Gleeson and Hancock 2001). It is clear that a full understanding of the relationship between performance and fitness requires evaluation of how performance in natural situations (i.e., ecological performance), as opposed to maximal (or sustained, steady-state) performance, is correlated with survival and reproduction.

Moving at maximal speed through a complex environment can be influenced by endogenous and exogenous factors. Therefore, the speed an animal chooses in a given context will be a compromise among such intrinsic and extrinsic factors (Wilson et al. 2015). Movement is energetically demanding and varies depending on the mode of locomotion an organism uses (Schmidt-Nielsen 1972; Taylor et al. 1982). Complex environments place a premium on maneuverability during movement. During legged locomotion, obstacles require organisms to slow down substantially to successfully navigate turns without loss of footing (Higham et al. 2001; Sathe and Husak 2015; Wynn et al. 2015). Successful navigation of obstacles requires the complex interplay among multiple neural circuits including the forebrain, brainstem, and spinal cord to integrate visuo-motor processes with other senses (Grillner and El Manira 2020). During locomotion, feedback processes between the visual system and the motor system permit accurate movement and avoidance of obstacles. Inevitably there is a delay in feedback such that during rapid locomotion the delay can result in compromised locomotor control (Grillner and El Manira 2020). Successfully navigating obstacles requires sufficient maneuverability, meaning that the animal must be able to overcome velocity in its current heading and redirect it toward a new heading (Jindrich and Full 1999). Larger body mass or faster locomotor speed in the current heading will reduce the angle that an animal can turn successfully. Therefore, increased speed can amplify the effect of delays in motor control and lead to reduced maneuverability. Consequently, the probability of making mistakes during legged locomotion should increase as locomotor speed increases because of a negative relationship between speed and maneuverability and between speed and accuracy (Wilson et al. 2015). In addition to balancing intrinsic constraints, aspects of the environment, extrinsic to the organism, can further influence the speeds chosen. For example, the slope, diameter, or coarseness of the substrate and the spatial distribution of obstacles (i.e., complexity of the habitat) can drastically impact locomotor speed (Losos and Sinervo 1989; Sinervo and Losos 1991; Tulli et al. 2012; Sathe and Husak 2015, 2018; Wheatley et al. 2018; Amir Abdul Nasir et al. 2017).

Given these constraints, successful locomotion in different ecological contexts likely will be optimized by balancing the costs and benefits of moving through the environment at different speeds (Wheatley et al. 2018). Successful foraging requires an animal to perceive the prey item, use controlled motor functions to navigate toward the prey item, capture it, and then consume it. While foraging, the speed chosen should be dependent upon the probability of encountering and detecting prey (i.e., prey abundance), the probability of encountering and detecting a predator, the energetic costs of movement (Wilson et al. 2015), and the costs of missed opportunities (i.e., missing prey). In addition to these factors, prey capture also should depend upon the relationship between speed, maneuverability, and motor control. The relative importance of these factors for successful foraging will also vary depending on the behavioral strategy adopted by the species. For example, speeds used by actively foraging species might be more constrained by the energetics of locomotion and visual detection of prey and predators. On the other hand, speeds chosen by sedentary foragers (i.e., sit-and-wait foragers) might be constrained more by trade-offs between speed and maneuverability and motor control, missed opportunities, and less so by energetics given that prey capture is typically an anerobic activity. For example, during the attack of a prey item, moving slowly would allow successful locomotion with few mistakes, but run the risk of prey escaping capture. An organism moving too rapidly during the attack would suffer costs associated with compromised maneuverability and motor control, which would likely lead to unsuccessful prey capture attempts. Therefore, one would predict that prey capture success would be maximized at intermediate speeds (Wilson et al. 2015). Indeed, cheetahs use moderate speeds optimizing maneuverability while hunting agile prey (Wilson et al. 2013). In the cases discussed above, the structure of the environment and the spatial orientation of obstacles will also mediate speeds. Complex habitats would render prey detection more difficult, but also might make prey capture more difficult because organisms would have to navigate obstacles during attack. In a more open environment, a predator also runs the risk of being detected by the prey prior to, or during, an attack favoring a longer attack distance and faster prey capture speed. In all, open habitats would be predicted to result in faster optimal prey capture speeds and higher prey capture success than cluttered habitats.

We experimentally manipulated habitat complexity in laboratory arenas to explore how extrinsic factors influence speeds chosen during prey capture and foraging success in the lizard *Sceloporus consobrinus*. *Sceloporus consobrinus* has a broad distribution in the south-central United States where it inhabits an extensive array of habitats ranging from forests to open grasslands (Powell et al. 2016). The species is typically regarded as a sit-and-wait forager (Perry 1999) that spends considerable time sedentary, scanning the environment for prey. Given its high abundance and broad flexibility in habitat preferences, this species (and related species) serves as an excellent model for ecological and physiological studies.

Because of trade-offs between speed and maneuverability, and between speed and motor control (i.e., accuracy), we tested the general hypothesis that habitat structure would have a significant impact on the speeds chosen by lizards during foraging trials. We placed lizards into foraging arenas that differed in the spacing of obstacles, measured the speeds used during foraging attempts, and studied how this variation in habitat structure influenced the probability of successful prey capture and consumption. Specifically, we predicted that (1) lizards would use slower speeds during foraging attempts in arenas with abundant obstacles than in open habitats; (2) because of reduced visibility in complex arenas, lizards would initiate prey capture attempts from shorter distances; (3) because of intrinsic constraints on speed, the probability of capturing a prey item will decline with increasing speed; and (4) increasing habitat complexity would change the relationship between prey capture speed and foraging success.

## Methods

In May and June 2018, we captured 29 adult lizards (snout–vent length (SVL) = 62.05 ± 1.32 mm, mean ± SE, range = 50–73 mm) from a forested location in Pulaski County, Arkansas. Our sample included 15 females and 14 males. All females were gravid upon capture but post-reproductive during experimental trials. We maintained lizards individually in arenas (50 cm × 35 cm × 33 cm; L × W × H) that included a substrate of play sand, a rock, or wooden block situated beneath a 60 W incandescent bulb, and a small portion of egg crate for a refuge. Room and basking lights were programed to supply a natural photoperiod (14:8 h, Light:Dark). We fed lizards a mixed diet of crickets and mealworms two to three times per week and provided water twice per day by spraying the inner walls of each arena.

**Fig. 1.**
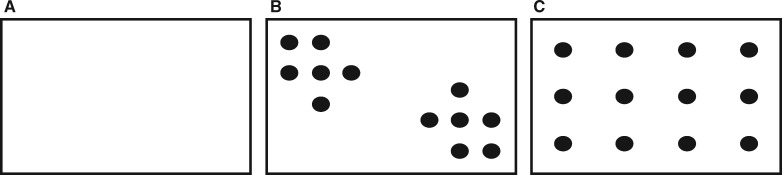
Diagrammatic representation of the arena design used in this study. Solid circles represent the arrangement of the wooden dowels in the arenas. (**A**) represents the OPEN arena, (**B**) the CLUSTERED arena, and (**C**) the UNIFORM arena. Arena dimensions are included in the text.

We conducted foraging trials in three custom-built experimental arenas of varying complexities arranged adjacent to each other (each arena 80 cm × 45 cm × 24 cm). Each arena contained a layer of sand and was heated by two 100 W incandescent bulbs suspended 50 cm above the sand surface. A gradient of habitat complexity was mimicked by altering the presence and configuration of obstacles (Fig. 1). In order, from least complex to most complex, we classified our arenas as OPEN, CLUSTERED, and UNIFORM. No obstacles were present in the OPEN arena, while 12 wooden dowels (2.5 cm in diameter) were arranged in both the CLUSTERED and UNIFORM arenas. Within the CLUSTERED arena, obstacles were arranged in opposite corners. Obstacles in the UNIFORM arena were evenly spaced throughout the arena. In both the CLUSTERED and UNIFORM arenas, the inclusion of obstacles reduced the arena area by approximately 58.9 cm^2^. Obstacles were spaced ∼7.5 cm apart in the CLUSTERED and the UNIFORM arenas (∼1.2 times the average lizard body length). We considered the UNIFORM arena a more complex environment than the CLUSTERED arena because obstacles were arrayed throughout the entire arena and lizards could not move far without encountering one. We used a repeated-measures design whereby each individual lizard was used in multiple trials in each arena type. To avoid confounding the order in which lizards experienced each trial, we placed animals in each arena in a random order and never in the same arena type in consecutive trials. We recorded foraging trails with hand-held HD video cameras (30 fps, GE DV1 1080p, General Electric), so we could analyze foraging attempts. Cameras were mounted to a custom-built stand 86 cm directly above the center of each arena. Prior to trials, we placed individuals in an arena for 10 min. After the 10-min acclimation period, we added three crickets to each arena and recorded lizard activity for 15 min. In all cases we introduced crickets of similar size. We attempted to ensure equal hunger/motivation among individuals in all trials by withholding food for 24 h prior to a trial. After each trial we measured lizards for body size (SVL and mass). SVL was measured to the nearest 0.5 mm using a ruler and mass was measured to the nearest 0.1 g with digital balance (Denver Instrument, Bohemia, New York). We initially reviewed video footage to determine a method for clearly identifying a foraging attempt. Each foraging attempt was preceded by the lizard orienting its head (and often body) facing the prey followed by direct locomotion toward the prey. Therefore, a foraging attempt was defined as any instance that the focal lizard oriented its head (and body) toward the prey and moved in an intentional, directed manner toward it. Because three crickets were used in each trial, many trials had multiple recorded foraging attempts; all of which were included in analyses. We examined video footage and used ImageJ (Schneider et al. 2012) to quantify the following variables for each attempt after calibrating each video to a known size standard (10 cm ruler) included on the floor of each arena (resulting video resolution = 15.8 pixels cm^−1^): (1) attack distance (cm), (2) prey capture speed (cm s^−1^), (3) capture success, and (4) consumption success. Attack distance (cm) was obtained by measuring the distance from the tip of the lizard’s snout, along a straight-line path, to the targeted prey item. Prey capture speed (cm s^−1^) was calculated by dividing the attack distance (cm) by the time, which was determined by counting the number of frames elapsed during the attack and dividing by the recording frame rate (30 fps). Finally, for each foraging attempt, we determined whether the prey item was captured and, once captured, if it was consumed.

## Statistical analyses

Attack distance data were strongly right-skewed. A natural log-transformation resulted in a normal distribution of the distance data. Speed data were not transformed. Because individual lizards were tested repeatedly in all three experimental arenas resulting in a crossed (i.e., multiple membership) design, we used mixed-effects models with trial number and individual ID as random effects. We tested the hypothesis that prey capture speeds and attack distance would differ among arena types (OPEN, CLUSTRED, and UNIFORM) using linear mixed effects models in the lmerTest package (Kuznetsova et al. 2017) of R v. 3.6.2 (R Core Team 2019).

We tested for variation among arena types in the probability of prey capture and consumption as a function of speed using generalized linear mixed models in the glmmTMB package in R (Brooks et al. 2017). Prey capture and prey consumption were coded as successful (1) or unsuccessful (0). As such these analyses assumed a binomially distributed error and used a logit link function. In these analyses we included speed, arena type (OPEN, CLUSTERED, UNIFORM), and the interaction between speed and arena type as fixed effects. As in previous analyses, trial number and individual ID were included as random effects to account for repeated measures of individuals within and among arena types. Statistical significance of each fixed effect was tested using likelihood-ratio tests. We conducted similar analyses by substituting ln-transformed initiation distance for speed as the dependent variable to test how the probability of prey capture and consumption varies as a function of initiation distance across arena types.

Successful foraging not only requires prey capture but also consumption of the prey item once captured. Therefore, prey capture alone might not be the best metric of foraging success. Additionally, the difference in the probability of prey capture and the probability of prey consumption might also vary with speed and represent a way to quantify how accuracy changes with speed. To conduct an analysis of foraging accuracy, we calculated the difference in model predictions describing the probability of capture and the probability of consumption as functions of speed. The resulting relationships describe the change in probability of success as speed increases. In other words, a prey item might be captured but then escape, and the probability of this occurring might increase as speed increases. In addition, the relationship between the probability of a prey item escaping after being caught and speed might vary depending on habitat (i.e., arena) structure. We explored these differences qualitatively (i.e., graphically).

## Results

**Fig. 2.**
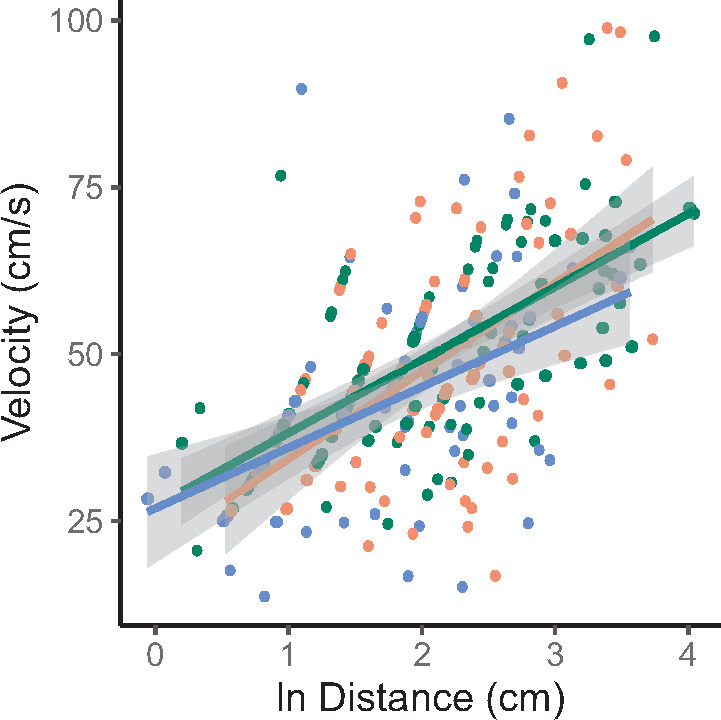
The relationship between foraging velocity (cm s^−1^) and attack initiation distance (cm) across all trials and foraging attempts. Orange dots and regression line denote foraging attempts in the CLUSTERED arena, green dots and line denote the OPEN arena, and blue dots and line denote the UNIFORM arena.

**Fig. 3.**
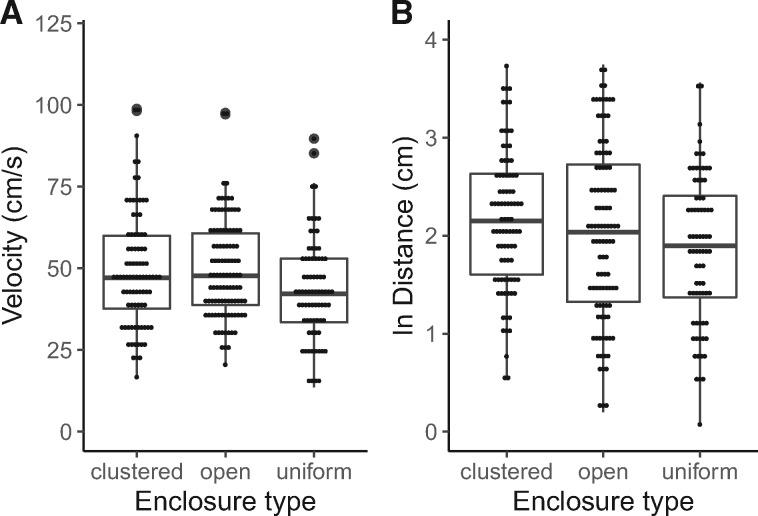
The effect of arena types on (**A**) foraging velocity (cm s^−1^) and (**B**) attack initiation distance (cm). Boxes represent the first quartile, median, and third quartile. Whiskers represent the 95% confidence intervals. All data represented in each box plot.

We obtained data from 261 total foraging attempts; 33% (*n* = 86) of all attempts occurred in the CLUSTERED arena, 39% (*n* = 102) from the OPEN arena, and 28% (*n* = 73) from the UNIFORM arena. Prey capture speed was positively correlated with attack initiation distance (*r* = 0.398, *P* < 0.001), and the relationship was similar across arena types (arena × distance interaction, F_2,244.37_ = 1.015, *P* = 0.364, Fig. 2). Across all trials, regardless of foraging success, prey capture speed showed minor variation across arena types (F_2,32.17_ = 2.620, *P* = 0.109), and distance traveled during a foraging attempt differed across habitat configurations (F_2,23.07_ = 4.312, *P* = 0.026, Fig. 3A, B). On average, lizards in the OPEN and CLUSTERED arenas traveled ∼15% faster than lizards in the UNIFORM arena (49.5 vs. 42.9 cm s^−1^), but those differences were not statistically different. Lizards in the CLUSTERED arena traveled on average 38.8% farther than lizards in the UNIFORM arena (11.8 vs. 8.5 cm; Tukey, *P* = 0.020), and those in the OPEN arena traveled 29.4% farther than those in the UNIFORM arena (11.0 vs. 8.5 cm, *P* = 0.122); the latter was not statistically significant.

**Fig. 4.**
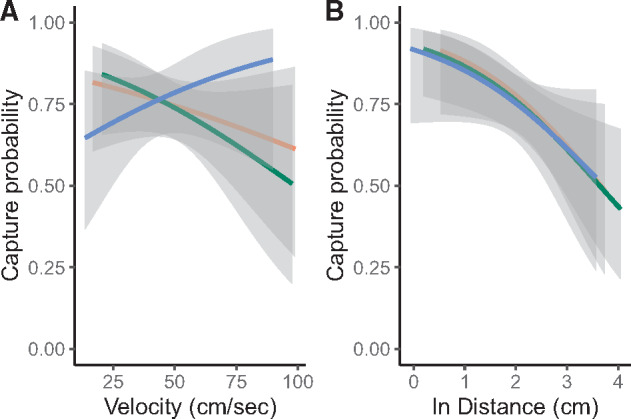
The effect of (**A**) foraging velocity (cm s^−1^) and (**B**) attack initiation distance (cm) on the probability of prey capture in each arena. The orange line denotes the CLUSTERED arena, the green line denotes the OPEN arena, and the blue line denotes the UNIFORM arena. The 95% confidence bands are shaded for each.

Neither prey capture speed nor arena type affected the probability of prey capture (speed: *X*^2^ = 0.804, *P* = 0.370; arena type: *X*^2^ = 0.056, *P* = 0.973). Likewise, the relationship between speed and the probability of prey capture was similar across arena types (*X*^2^ = 3.138, *P* = 0.208, Fig. 4A). The probability of prey capture declined with increasing attack initiation distance (*X*^2^ = 15.137, *P* < 0.001); and the relationship was similar among arena types (*X*^2^ = 0.042, *P* = 0.979, Fig. 4B). Finally, the mean probability of prey capture was also similar among arenas (*X*^2^ = 0.110, *P* = 0.946).

**Fig. 5.**
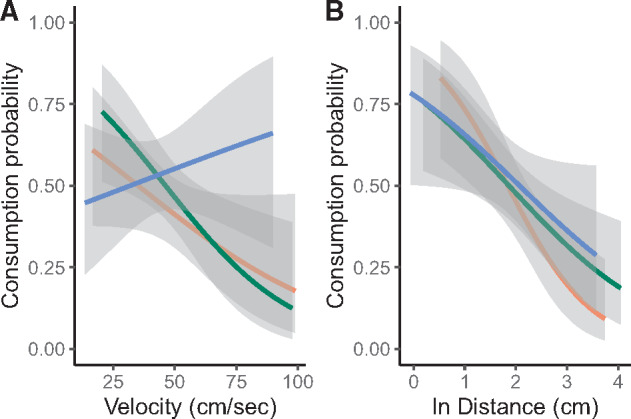
The effect of (**A**) foraging velocity (cm s^−1^) and (**B**) attack initiation distance (cm) on the probability of prey consumption in each arena. The orange line denotes the CLUSTERED arena, the green line denotes the OPEN arena, and the blue line denotes the UNIFORM arena. The 95% confidence bands are shaded for each.

In contrast to prey capture, the probability of prey consumption generally declined with increasing prey capture speed (*X*^2^ = 4.613, *P* = 0.032), and this relationship differed among arena types (*X*^2^ = 6.530, *P* = 0.038). Specifically, prey consumption success strongly declined with increasing speed for lizards in the OPEN and CLUSTERED arenas, but it was unaffected or increased slightly with speed for lizards in the UNIFORM arena (Fig. 5A). Similarly, speed during successful foraging attempts was slower than unsuccessful attempts in the OPEN (F_1,97.03_ = 6.876, *P* = 0.010) and CLUSTERED (F_1,83.981_ = 3.736, *P* = 0.057) arenas, but speed was similar in the UNIFORM arena (F_1,62.28_ = 1.019, P = 0.316). In all three arenas prey consumption probability declined markedly within increasing attack initiation distance (*X*^2^ = 20.763, *P* < 0.001) and similarly among arena types (*X*^2^ = 1.807, *P* = 0.405, Fig. 5B).

**Fig. 6.**
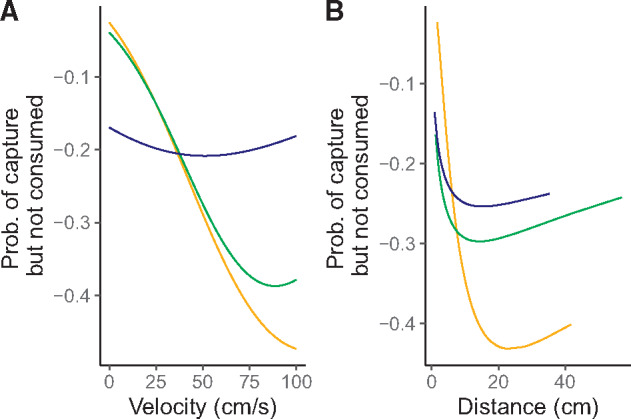
Relationships between foraging accuracy and (**A**) velocity (cm s^−1^) and (**B**) attack initiation distance (cm). Foraging accuracy was calculated as the difference between the model predicted probabilities of prey consumption and prey capture (Figs. 4 and 5). A value of zero means that the probability of prey capture and prey consumption are equivalent. Increasingly negative numbers are interpreted as increasing probability that a captured prey item escapes the predator’s grasp. The orange line denotes the CLUSTERED arena, the green line denotes the OPEN arena, and the blue line denotes the UNIFORM arena.

In the OPEN and CLUSTERED arenas, foraging accuracy appeared to be very sensitive to prey capture speed such that the probability of a prey item escaping, once captured, increased (Fig. 6A; shown by an increasing difference between the probabilities of capture and consumption as speed increased). On the other hand, foraging accuracy appeared relatively invariant across prey capture speeds for lizards in the UNIFORM arena. Foraging accuracy also was sensitive to the distance traveled during a foraging attempt; although, in this case, foraging accuracy of lizards in the CLUSTERED arena declined most rapidly with distance (Fig. 6B).

## Discussion

We examined key elements of foraging behavior (i.e., prey capture speed and attack distance) and their impact on foraging success across varying habitat configurations. We predicted that in more complex habitats, lizards would use both slower speeds and attack prey from shorter distances. Additionally, we expected that increased speeds would result in a decreased probability of successfully capturing and consuming prey caused by limitations on maneuverability and accuracy at high speeds. We also hypothesized that the association between speed and foraging success would be impacted by increasing habitat complexity.

Our results were generally consistent with our predictions. We found that lizards selected ∼15% slower speeds in our most cluttered habitat (UNIFORM). Fast speeds often inhibit an animal’s ability to maneuver around obstacles without slipping, so animals might use slower speeds during prey capture in habitats with abundant (and closely spaced) obstacles in order to increase their chances of success. Indeed, several studies forcing animals to sprint and maneuver around obstacles or corners of varying turning angles found that speed was modulated to slower levels allowing successful navigation (Higham et al. 2001; Sathe and Husak 2015; Wynn et al. 2015). Habitats with abundant, and evenly spaced, obstacles also would be predicted to obscure visibility more so than those with clustered obstacles or those lacking them altogether. We found that lizards tended to travel greater distances during foraging attempts in our two most open arenas (OPEN and CLUSTERED), suggesting that evenly spaced obstacles may reduce visibility of prey to the predator, or that the predator must attack prey from greater distance with faster speed to avoid being detected by the prey. Consistent with this observation, lizards in the OPEN arena also tallied the largest proportion of total foraging attempts (*n* = 102, 39%), followed by lizards from the CLUSTERED arena (*n* = 86, 33%), and finally the UNIFORM arena (*n* = 73, 28%).

Variation in mean prey capture speed and mean attack initiation distance, although predicted, does not tell the complete story of foraging success. Successful foraging requires an animal to perceive the prey item, use controlled motor functions to navigate toward the prey item, capture it, and then consume it. Moving from too great a distance might allow the prey to see the approaching predator and avoid capture and consumption. Moving too quickly can reduce the accuracy of the predator, resulting in the prey escaping after capture. Long distance foraging attempts might also require the predator to successfully navigate obstacles during the attack. Thus, we made two predictions. First, intrinsic constraints on speed would cause the probability of foraging success to decline with increasing speed and distance. Second, structural habitat variation would modify the relationship between foraging success and speed and distance. Interestingly, we found that the distribution of obstacles in arenas did not statistically impact the probability of successful prey capture, nor was prey capture speed correlated with prey capture overall. Although we did not detect a statistical relationship, inspection of Fig. 3A illustrates that lizards from the OPEN and CLUSTERED arenas had reduced prey capture success when moving at faster speeds; whereas lizards from the UNIFORM arena trended in the opposite direction. It is likely that prey also is constrained by the spacing of obstacles, making capture easier, even for a fast-moving predator. More detailed analysis, also measuring prey escape behavior (and escape speed), would be necessary to more fully determine this possibility. We were unable to confidently measure prey escape speed in this study given the video recording speed used.

Foraging success is ultimately determined not only by capturing prey, but also by consuming it. Therefore, prey capture alone is likely not the best metric of foraging success. Consistent with our predictions, the probability of prey consumption was negatively correlated with prey capture speed overall, and this relationship differed among arenas. Specifically, prey consumption probability declined rapidly with increasing prey capture speed in OPEN and CLUSTERED arenas, but did not vary strongly with speed in the UNIFORM arena (Fig. 5A). Indeed, foraging attempts ending with successful consumption were approximately 16% slower than those where the prey was not consumed in both the OPEN and CLUSTERED arenas. This was not the case for attempts in the UNIFORM arena. In all arenas, whether considering prey capture or consumption, the probability of foraging success declined significantly (and similarly) with attack initiation distance. The difference between the probability of prey capture and consumption was primarily mediated by variation in prey capture speed.

The tradeoff between speed and control/accuracy constrains all animal movements and the neural basis of such movement decisions are beginning to be understood (Dean et al. 2007; Bogacz et al. 2010; Wenzlaff et al. 2011). In goal-directed movements, such as during predator–prey interactions, increasing speed leads to a decline in accuracy of movement and decreased success (Clemente and Wilson 2016). Because prey capture and prey consumption probabilities differed in their relationships with foraging velocity, it appears that the accuracy of the attack was highly sensitive to the speed used during attack, as expected. We attempted to explore this relationship by calculating the difference between predicted consumption probability and capture probability and plotting this across the range of prey capture speeds. Predator accuracy declined markedly with increasing speed in the OPEN and CLUSTERED arenas, whereas accuracy remained fairly constant in the UNIFORM arena (Fig. 6). Therefore, as the speed of an attack increased, it was much more likely that the captured prey item would escape in the former two arenas. In these cases, it was typical that the lizard captured the cricket by a leg, which was then autotomized by the cricket, permitting its escape.

Consistent with studies conducted in the field, lizards in our experiment used speeds while foraging that were considerably slower than maximal speeds attainable. Robinson and Gifford (2018) recently measured maximal sprinting speed in *S. consobrinus* across a range of ecologically relevant temperatures. Maximal sprinting speed at 34°C was 1.81 ± 0.05 m s^−1^. The minimum and maximum prey capture speeds measured in our study were 8% (0.15 m s^−1^) and 55% (1.0 m s^−1^) of the maximum capacity, respectively, and mean speeds were ∼23–27% of maximum capacity. Thus, the speeds measured in this study are comparable to those reported for other species in natural settings (Husak and Fox 2006), suggesting that our results are ecologically relevant. While foraging, animals are predicted to select speeds that represent a balance between being fast enough to capture a prey item and slow enough to accurately navigate their environment and avoid costly mistakes (Wilson et al. 2015). Results presented here indicate that the structure of the habitat, particularly the spacing of obstacles, can substantially modify the relationship between speed and foraging success. Ultimately, it appears that success is primarily constrained by intrinsic properties when ample foraging space is available, but environmental conditions have a greater impact on foraging success in “cluttered” habitats than in open habitats. The present work provides an empirical test of theoretical predictions about optimal movement speeds in animals and provides a step forward in understanding how animals select speeds in nature.

More than 30 years ago, Hertz et al. (1988) and Pough (1989) encouraged researchers to study how organisms actually perform in nature. The last two decades have seen a dramatic increase in studies examining performance in different ecological contexts, often made possible by field portable high-speed video equipment (Irschick and Losos 1998; Jayne and Ellis 1998; Irschick and Jayne 1999; Irschick 2003; Irschick et al. 2005; Husak and Fox 2006; Muniz-Pagan et al. 2012). This experimental study suggests that increasing habitat complexity can influence foraging success and the speeds animals use when foraging. A similar approach to ours, but in a field context, using measurements of microhabitat structure would be a valuable addition to our understanding of animal foraging and ecological performance. We encourage researchers to consider such an endeavor.

## Author contributions

M.E.G. analyzed the data, conceived the ideas, and designed the methodology. D.R.A. contributed to the data collection. M.E.G. and D.R.A. led the writing of the manuscript.

## Notes

The authors contributed critically to early drafts and gave final approval for publication.
